# Dissecting the mammary gland one cell at a time

**DOI:** 10.1038/s41467-018-04905-2

**Published:** 2018-06-26

**Authors:** Simona Cristea, Kornelia Polyak

**Affiliations:** 1 Department of Biostatistics and Computational Biology, Dana-Farber Cancer Institute, Boston, 02215 MA USA; 2 Department of Biostatistics, Harvard T. H. Chan School of Public Health, Boston, 02115 MA USA; 3 Department of Stem Cell and Regenerative Biology, Harvard University, Cambridge, 02138 MA USA; 4 Department of Medical Oncology, Dana-Farber Cancer Institute, Boston, 02215 MA USA; 5 Department of Medicine, Harvard Medical School, Boston, 02115 MA USA; 6 Harvard Stem Cell Institute, Cambridge, 02138 MA USA

## Abstract

Dissecting cellular differentiation hierarchies in the mammary gland is a prerequisite for understanding both normal development and malignant transformation during tumorigenesis and tumor cell-of-origin. To achieve these goals, several recent papers utilized single cell RNA-seq and lineage tracing to improve our understanding of the composition of the mammary epithelium at different developmental stages.

The mammary gland is a branching epithelial structure composed of ducts and alveoli^1^. It is a unique organ that completes its development and differentiation during puberty and adulthood, orchestrated by ovarian and pituitary hormones, to fulfill its main function of milk production during lactation. The mammary epithelium consists of two differentiated cell types organized into two cell layers, an inner layer of luminal epithelial and an outer layer of myoepithelial cells in direct contact with the basement membrane (Fig. 1a). Functional studies employing transplantation of tissue pieces, cell populations sorted for various cell surface markers, or single cells, as well as lineage tracing using cell type-specific promoters have demonstrated the existence of bipotential mammary epithelial stem cells and lineage-committed luminal and myoepithelial progenitors both in human and mouse^2^. These studies have, however, yielded differing results. Some have suggested that bipotential stem cells are only present during development, and in adulthood the mammary gland is maintained by lineage-committed progenitors^3^, while others proposed the emergence and expansion of some progenitors only during pregnancy^4^. To decipher mammary epithelial cell differentiation hierarchies in a comprehensive and unbiased manner, several groups applied single cell RNA-seq (scRNA-seq) to the mammary gland in human^5^ and in mice^6,7^, while another study used lineage tracing to follow the fate of Blimp1^+^ stem cells^8^.

**Fig. 1:**
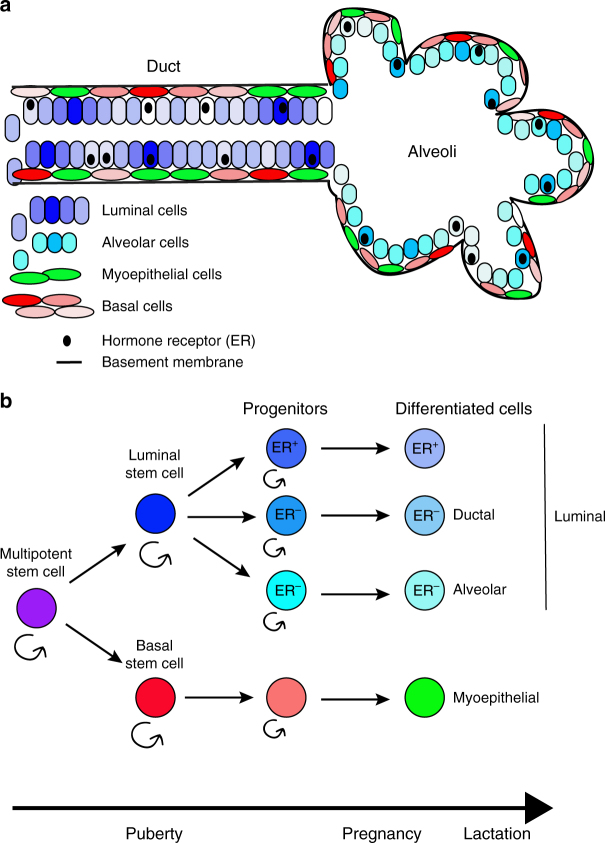
Simplistic model of mammary epithelial cell differentiation hierarchy. **a** Schematic outline of a ductal-alveolar unit with location of the various cell types indicated. **b** A putative map of mammary epithelial cell differentiation. A multipotent stem cell present during development gives rise to luminal epithelial and basal stem cells, which further divide into luminal and basal progenitors during puberty. Ductal and alveolar hormone-receptor negative progenitors are distinct lineages and there is also a separate hormone receptor positive luminal lineage

Defining the cellular composition of a solid organ is a challenging task requiring optimized methods to ensure reproducibility. First, the tissue has to be dissociated into single cells fairly rapidly, to minimize perturbation of cellular features. Second, the accurate detection of minor subpopulations, present as low as 1 in a 1000 cells frequency, requires the portrayal of thousands of cells. The characterization of the mammary gland is even more challenging as it undergoes dramatic changes during postnatal development and more subtle variations during menstrual/estrus cycles in response to ovarian and pituitary hormones.

To tackle these challenges, Pal et al.^7^ characterized the mouse mammary epithelium at the single cell level at four developmental stages, pre-puberty, mid-puberty, adult virgin, mid-pregnant, and also at different phases of the estrus cycle. Similarly, Bach et al.^6^ profiled mammary epithelial cells (MECs) in mice at four developmental stages: adult virgin, mid-gestation pregnant, day 6 lactating, and 11 days post involution. The two groups have largely overlapping, but also some seemingly discordant findings, potentially due to differences in cell purification and data analysis procedures. Pal et al. concluded that basal gene expression occurs throughout all developmental stages, with a particularly distinct and homogeneous profile in the pre-pubertal gland, whereas luminal expression is only detected at puberty through adulthood. This suggests that there may be a hormone-responsive luminal progenitor that subsequently gives rise to both hormone-responsive and non-responsive luminal epithelial cells or that a subset of basal cells responds to ovarian hormones and generates luminal progeny. The authors also identified one basal, and several distinct luminal cellular expression clusters; some were expected based on prior studies like mature luminal (ML) cells and luminal progenitors (LP), while others were novel like a luminal intermediate (a transit population between ML and LP cells), and a mixed-lineage subpopulation expressing both luminal and basal markers.

Bach et al.^6^ reached somewhat differing conclusions finding that mammary epithelial cells display a differentiation continuum rather than clearly defined clusters, suggesting that a common luminal progenitor cell gives rise to intermediate, restricted alveolar, and hormone-sensitive progenitors. The authors divided the cells into 11 luminal and 4 basal clusters (based on the expression of known marker genes), proposing a putative differentiation tree. The basal cluster was further subdivided into differentiated myoepithelial, and stem cell-like basal, and Procr^+^ cells, while the luminal compartment was classified into hormone-sensing cells (both progenitors and terminally differentiated) and cells expressing low levels of hormone receptors. Using diffusion maps, the authors reconstructed the differentiation states in the mammary gland showing luminal and basal clusters clearly segregated but with states transitioning between the secretary alveolar lineage and hormone-sensing luminal cells implying origination from the same progenitor. The authors provide an interactive online presentation of the expression data, making it available to the community and allowing the easy interrogation of the expression of particular genes of interest. Nevertheless, a limitation of this study is that too few basal cells were captured such as to be able to define a hierarchy. Contrary to Pal et al.^7^, no mixed-lineage population with both basal and luminal markers was detected, either because this population is not present, or as a technical consequence of the different sorting and data analysis strategies employed.

Nguyen et al.^9^ focused on the normal human breast and analyzed breast epithelial cells isolated from reduction mammoplasties of healthy adult pre-menopausal women. Cells were sorted prior to RNA-seq based on known cell surface markers into luminal and basal subsets (CD49f and EPCAM, respectively). The analysis identified three main clusters essentially in line with previously described groups of basal/mammary stem cells (CD49f^hi^EPCAM^−^), luminal progenitors (L1:CD49f^+^EPCAM^+^), and mature luminal (L2:CD49f^−^EPCAM^+^) cells. Pseudotemporal reconstruction of differentiation trajectories produced one continuous lineage connecting the basal and the two differentiated luminal branches. The authors further subdivided these three main clusters into subclusters showing some inter-individual variability, which were interpreted as cell states, not distinct cell types. The three basal subclusters were characterized as high in inflammatory mediators, myoepithelial cell markers, and specific keratins. An intriguing finding of this study is the detection of replicating cells in all three main clusters (Basal, L1, and L2) based on the expression of proliferation markers in the scRNA-seq data (confirmed by immunofluorescence). This finding implies that each cluster may be maintained by its own stem/progenitor cell population with proliferative capacity.

Elias et al.^8^ applied lineage-tracking to examine the relationship between Blimp1-expressing cells and previously described luminal progenitor subpopulations. Blimp1 is a repressor that governs cell fate decisions in embryonic and adult tissues and is robustly induced in alveolar cells during pregnancy^10^. The authors identified very rare Blimp1^+^ lineage-restricted, unipotent luminal progenitor cells that maintain their identity through adult life, display extensive self-renewal capacity, and play essential roles in duct formation, homoeostasis and alveologenesis during pregnancy. Specifically, Blimp1^+^ cells give rise to highly proliferative Elf5^+^ERα^-^PR^−^luminal progenitors that expand during pregnancy to promote alveologenesis, and the Blimp1^+^ cell population present during pregnancy is derived exclusively from the rare Blimp1^+^ cells originally labelled during puberty. The experiments presented show no evidence that Blimp1^+^ progenitor cells emerge de novo in the post-natal gland, but rather that Blimp1^+^ cells are maintained as a constant pool of long-lived alveolar progenitors throughout pregnancy-associated mammary gland remodeling. These data also suggest that hormone-responsive luminal epithelial and hormone receptor-negative alveolar cells represent different lineages, which is in line with results of prior lineage tracking studies using different markers to follow the cells^11–13^.

The studies briefly discussed here represent valuable contributions to our understanding of the cellular composition of the mammary epithelium at different reproductive stages in mice and human, providing a useful resource easily accessible to the public. Since all three scRNA-seq papers used different types of sorting for the cell populations profiled and focused on epithelial cells, it would be important to compare these results to that of the Mouse Cell Atlas^14^, which profiled all cells of the mammary gland without any purification, or be used by the Human Cell Atlas^15^ initiative to provide informative experimental design frameworks. Overall both the scRNA-seq and the lineage tracking data support a model whereby in the post-pubertal mammary gland differentiated luminal epithelial and myoepithelial cells are maintained by lineage-restricted progenitors and bipotential cells are present through embryonic development up to puberty (Fig. 1b). Lineage tracking also suggests that hormone-responsive luminal and non-responsive alveolar cells may also represent distinct lineages. Lineage-restricted progenitors appear to be established during puberty and some of their progeny expand during pregnancy. This model is consistent with human epidemiologic data demonstrating that puberty is a critical stage for establishing life-long breast cancer risk. Nevertheless, additional work is necessary in terms of sequencing of more cells, sampled in an unbiased manner, at higher depth and linking the molecular profiles with functional data obtained in unperturbed experimental systems.
